# MONSTROUS: a web-based chemical-transporter interaction profiler

**DOI:** 10.3389/fphar.2025.1498945

**Published:** 2025-02-26

**Authors:** Mohamed Diwan M. AbdulHameed, Souvik Dey, Zhen Xu, Ben Clancy, Valmik Desai, Anders Wallqvist

**Affiliations:** ^1^ Department of Defense Biotechnology High Performance Computing Software Applications Institute, Telemedicine and Advanced Technology Research Center, Defense Health Agency Research and Development, Medical Research and Development Command, Frederick, MD, United States; ^2^ The Henry M. Jackson Foundation for the Advancement of Military Medicine, Inc., Bethesda, MD, United States

**Keywords:** transporter profiler, graph convolutional neural network, ABC transporters, SLC transporters, chemical transporter interactions, transporter screening

## Abstract

Transporters are membrane proteins that are critical for normal cellular function and mediate the transport of endogenous and exogenous chemicals. Chemical interactions with these transporters have the potential to affect the pharmacokinetic properties of drugs. Inhibition of transporters can cause adverse drug-drug interactions and toxicity, whereas if a drug is a substrate of a transporter, it could lead to reduced therapeutic effects. The importance of transporters in drug efficacy and toxicity has led regulatory agencies, such as the U.S. Food and Drug Administration and the European Medicines Agency, to recommend screening of new molecular entities for potential transporter interactions. To aid in the rapid screening and prioritization of drug candidates without transporter liability, we developed a publicly available, web-based transporter profiler, MOlecular traNSporT inhibitoR and substrate predictOr Utility Server (MONSTROUS), that predicts the potential of a chemical to interact with transporters recommended for testing by regulatory agencies. We utilized publicly available data and developed machine learning or similarity-based classification models to predict inhibitors and substrates for 12 transporters. We used graph convolutional neural networks (GCNNs) to develop predictive models for transporters with sufficient bioactivity data, and we implemented two-dimensional similarity-based approach for those without sufficient data. The GCNN inhibitor models have an average five-fold cross-validated receiver operating characteristic area under the curve (ROC-AUC) of 0.85 ± 0.07, and the GCNN substrate models have an average ROC-AUC of 0.79 ± 0.08. We implemented the models along with applicability domain calculations in an easy-to-use web interface and made it publicly available at https://monstrous.bhsai.org/.

## 1 Introduction

Transporters are membrane proteins that are essential for normal physiological functioning of the human body and play a key role in transport of endogenous metabolites, signaling molecules, nutrients, drugs, and toxic chemicals (International Transporter Consortium et al., 2010; Nigam, 2015). The ATP-binding cassette (ABC) family and the solute carrier (SLC) family are two major superfamilies of transporters that are widely expressed in biological membranes of various tissues, including the liver, kidney, and brain (Giacomini et al., 2022; Galetin et al., 2024). Bile salt export pump (BSEP), P-glycoprotein (Pgp), breast cancer resistance protein (BCRP), and multidrug resistance protein (MRP) are well-known examples of ABC transporters (Sajid et al., 2023). Organic anion transporting polypeptide (OATP)-1B1, OATP1B3, organic cation transporter (OCT)-1, and multidrug and toxin extrusion transporter (MATE)-1 are well-known examples of SLC transporters (Turkova and Zdrazil, 2019). These transporters play a role in the influx/uptake of drugs as well as their efflux out of the cells and have the potential to impact the pharmacokinetic properties of drugs (Liu and Sahi, 2016). For example, if a small-molecule drug is a substrate of an efflux transporter, it can lead to reduced therapeutic effects, and if it is an inhibitor of transporters, it can cause clinically relevant drug-drug interactions that affect treatment outcomes (Gordon et al., 2016; Liu et al., 2017; Ciuta et al., 2023; Galetin et al., 2024).

Inhibition of transporters can also causally lead to adverse outcomes. For example, chemical-induced inhibition of BSEP, an ABC family transporter, is now recognized as the molecular initiating event for the cholestasis adverse outcome pathway (Vinken et al., 2013). Transporters are also a critical component of the blood-brain barrier (BBB) (Rankovic, 2015; Iorio et al., 2016). Any compound that acts as a substrate for ABC transporters, such as Pgp and BCRP, is effluxed out of the BBB, leading to poor bioavailability in the central nervous system (Iorio et al., 2016; Bellettato and Scarpa, 2018).

On the other hand, transporters can also be considered as therapeutic targets in cancer treatment (Silbermann et al., 2020). ABC transporter inhibitors, including inhibitors of Pgp, BCRP, and MRP, increase the efficacy of anti-cancer treatments and have the potential to serve as an adjuvant to cancer chemotherapy (Pena-Solorzano et al., 2017; Li et al., 2023). Many medicinal chemistry efforts to develop new inhibitors targeting ABC transporters have been reported in the literature (Pan et al., 2013; Munagala et al., 2014; Kohler and Wiese, 2015; Silbermann et al., 2020; Namasivayam et al., 2021; Kwon et al., 2023; Li et al., 2023).

More recently, regulatory agencies, such as the U.S. Food and Drug Administration (FDA) and the European Medicines Agency, have provided recommendations that require screening of new molecular entities for potential transporter interactions (Galetin et al., 2024). However, experimental screening to identify transporter inhibitors/substrates for the large set of chemicals evaluated in the early stages of drug discovery is resource-intensive and time-consuming. Computational approaches provide an alternative to experimental screening (AbdulHameed et al., 2016; Luechtefeld et al., 2018). Such computational methods can be either structure-based or ligand-based. Ligand-based approaches only require the structure of the compound and its activity value against particular transporters. Most of the previous transporter computational modeling studies have been predominantly ligand-based analyses (Ekins et al., 2002; Pan et al., 2013; Montanari and Ecker, 2015; Schyman et al., 2016; Schlessinger et al., 2018; Turkova et al., 2019; McLoughlin et al., 2021; Lane et al., 2022; AbdulHameed et al., 2023; Kong et al., 2023; Nigam et al., 2024).

Ligand-based models for chemical-transporter interactions can either predict the potential of a chemical to act as an inhibitor, reducing transporter activity, or as a substrate, meaning the chemical itself can be transported (Schlessinger et al., 2018). Such models can be local, focusing on a particular chemical series, or global, covering a diverse set of compounds (Montanari and Zdrazil, 2017). Typically, local models are more suited for hit optimization of a particular chemical series, and global models cover a wider range of chemicals and are suitable for virtual screening (Montanari and Zdrazil, 2017; Schlessinger et al., 2018). Predicting both inhibitory potential and substrate activity is crucial for successful drug development, as highlighted above. As part of the series of papers from the International Transporter Consortium, Schlessinger et al. provide a comprehensive summary of various computational modeling studies of drug-transporter interactions, using techniques such as multiple linear regression, k-nearest neighbors, support vector machines, random forest, and Bayesian classification approaches (Schlessinger et al., 2018). More recent literature reviews provide a detailed summary of the computational modeling studies reported for various ABC and SLC transporters (Turkova and Zdrazil, 2019; Kong et al., 2023).

While computational studies typically focus on one transporter of interest, simultaneously predicting the potential of a chemical to interact with a range of transporters would be more useful. Sedykh et al. built the first grouped model for predicting the inhibition and substrate potential of chemicals for a range of transporters expressed in the gastrointestinal tract (Sedykh et al., 2013). Aniceto et al. and Shaikh et al. have reported studies on developing substrate models (Aniceto et al., 2016; Shaikh et al., 2017). Most of the computational machine learning models reported so far share the models as a supplementary file or on GitHub and allow only programmatic access. However, there is a need for an easy-to-use web interface that enables experimental research groups and others to rapidly screen their compounds of interest for transporter liability. The Ecker lab took a first step towards this and created the Vienna LiverTox Workspace as a web tool to predict chemical interactions with transporters known to be critical in the liver (Montanari et al., 2019). This is the culmination of their earlier works in this area, and their tool has inhibitor models for seven transporters and substrate models for five transporters (Pinto et al., 2012; Poongavanam et al., 2012; Kotsampasakou et al., 2015; Kotsampasakou and Ecker, 2017; Montanari et al., 2019). They reported cross-validated balanced accuracies in the range of 0.64–0.88 (Montanari et al., 2019). However, the batch screening option, for screening a large number of compounds, of the Vienna LiverTox tool is not publicly available.

Previous transporter models reported so far have primarily relied on molecular descriptors or fingerprints to represent compounds during model development. Graph convolutional neural networks (GCNNs) offer an alternative approach for model building that has not yet been explored in transporter model development. GCNNs represent a recent advancement in cheminformatics that allows automated learning of molecular structures in contrast to traditional fingerprint-based approaches that require predefined sets of chemical substructures (Liu et al., 2019; Yang et al., 2019). A comprehensive evaluation using benchmark datasets has demonstrated that the GCNN approach performs better than the traditional fingerprint- and descriptor-based approaches (Yang et al., 2019).

**FIGURE 1:**
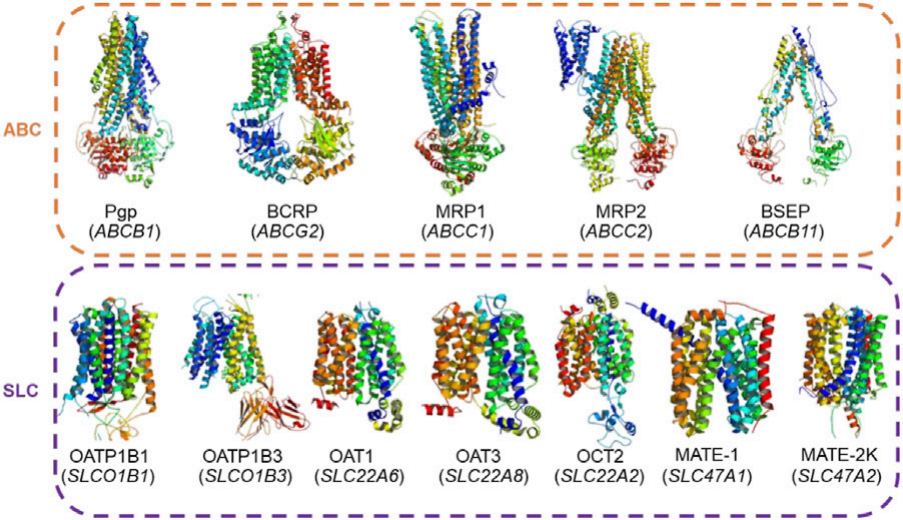
Actual structures of the 12 transporters used in this study and their gene symbols (given in parentheses). These transporters belong to the ATP-binding cassette (ABC) and solute carrier (SLC) transporter superfamilies and are recommended by regulatory agencies for testing during drug development.

In this work, we created a publicly available web tool for chemical-transporter interaction prediction, MOlecular traNSporT inhibitoR and substrate predictOr Utility Server (MONSTROUS) (https://monstrous.bhsai.org/). We developed predictive models for 12 transporters recommended by regulatory agencies for screening during the early drug discovery stage (Figure 1). Our tool makes separate predictions for the potential of a chemical to be either a transporter inhibitor or a substrate, resulting in a total of 24 models. We adopted a hybrid approach during tool creation that allowed us to comprehensively cover screening of these transporters in one tool. When sufficient bioactivity data were available, we utilized the GCNN approach for model building, and in the other cases, we developed two-dimensional (2D) similarity-based screens. Six of the 12 inhibitor models and three of the 12 substrate models are GCNN-based models, and the rest use the similarity-based screening approach. We implemented the sum of distance-weighted contributions (SDC) approach to define the applicability domain for all the models (Liu and Wallqvist, 2019). We evaluated our models using cross-validation analyses. We also show an example analysis using a widely explored set of kinase inhibitors. Overall, the developed tool will aid in the rapid screening and prioritization of countermeasure/drug candidates without transporter liability.

## 2 Methods

### 2.1 Dataset and pre-processing

We utilized publicly available datasets that were collected either from previously published papers or from bioactivity databases, such as ChEMBL, BindingDB, and Metrabase, for model building and evaluation (Mak et al., 2015; Gilson et al., 2016; Jiang et al., 2020; Zdrazil et al., 2024). For the 12 transporters used in this work, we collected separate data for inhibitors and substrates. Supplementary Table S1 provides a detailed list of data sources for each transporter in this study. We pre-processed the simplified molecular-input line-entry system (SMILES) using the ChEMBL structure pipeline, which is comprised of three functions, namely, checker, standardizer, and salt strip, to check for validity of chemical structure, format compounds to standardized conventions, and strip salts, respectively (Bento et al., 2020). We created a final pre-processed dataset for model building after evaluation and removal of duplicate compounds. The final set of pre-processed data used for model building is provided on the GitHub page (https://github.com/bhsai/monstrous).

### 2.2 Model building

We utilized Chemprop, an open-source software, to develop the GCNN models (Yang et al., 2019; Heid et al., 2024). This program uses a directed message-passing neural network (D-MPNN) to generate molecular features (Heid et al., 2024). The D-MPNN models the molecular structure as a graph, where atoms are represented as nodes and bonds as edges. Each node and edge is assigned a feature vector that captures the characteristics of the corresponding atom and bond. The Chemprop approach involves two phases: the message-passing phase and the readout phase (Yang et al., 2019). In the message-passing phase, the D-MPNN iteratively refines the atomic- and bond-level features based on information from neighboring nodes and edges during each convolution operation. Ultimately, in the readout phase, the compound’s learned representation is generated using an aggregation function that combines the final updated features at both the atom and bond levels (Heid and Green, 2022). This learned representation is then fed into a feed-forward neural network, which uses it as the input feature vector to predict the compound’s activity. In this approach, the molecular representation is automatically learned by the program, eliminating the need for pre-defined chemical fingerprints.

The program processes a list of SMILES strings and their corresponding activity values, which are provided as 1 for active molecules and 0 for inactive ones, in a CSV format. In this study, we selected “classification” as the modeling type and used five-fold and 10-fold cross-validations for the “number of folds” option. Each run involved developing 10 ensemble models over 30 epochs. For the remaining model development parameters, we used the default values: a depth value of three, i.e., the number of message-passing steps in D-MPNN, the ReLu activation function, 300 hidden neurons, and two layers for the feed-forward neural network.

### 2.3 Performance evaluation

We carried out five-fold and 10-fold cross-validations as well as a scaffold split-based validation to understand model performance. In the five-fold cross-validation procedure, we split the dataset into five groups and left one group out; subsequently, we used the model built from the compounds in the remaining four groups to predict the compounds in the left-out group. Once we completed this prediction cycle by leaving out each of the five groups, we calculated the model evaluation parameters: the receiver operating characteristic (ROC) area under the curve (AUC) and the Matthews correlation coefficient (MCC). This approach of leaving out groups is commonly used during model development to evaluate the model’s ability to predict the activity of compounds not seen during training and assess its performance on unseen data. In the 10-fold cross-validation, we repeated the same process by dividing the data into 10 groups and leaving out one group. The results presented here are averages across cross-validation folds. Such a cross-validation study summarizes generalizability and robustness of the model. We also performed a scaffold split and created training and test sets. We developed the model, as described above, using the scaffold-split training data and evaluated the performance using the scaffold-split test data. We calculated the following metrics: sensitivity (also known as the recall or true positive rate), the ability to correctly predict positive results; specificity (also known as the true negative rate), the ability to correctly predict negative results; accuracy, the total percentage correctly predicted; and MCC. These parameters are defined as follows (Equations 1–4):

$$\text{Sensitivity}=\frac{\text{TP}}{\text{TP}+\text{FN}}$$ (1)

$$\text{Specificity}=\frac{\text{TN}}{\text{TN}+\text{FP}}$$ (2)

$$\text{Accuracy}=\frac{\text{TP}+\text{TN}}{\text{TP}+\text{TN}+\text{FP}+\text{FN}}$$ (3)

$$\text{MCC}=\frac{\text{TP}.\text{TN}\;‐\;\text{FP}.\text{FN}}{\sqrt{\left(\text{TP}+\text{FP}\right).\left(\text{TP}+\text{FN}\right).\left(\text{TN}+\text{FP}\right).\left(\text{TN}+\text{FN}\right)}}$$ (4)

where TP represents true positive, TN denotes true negative, FP represents false positive, and FN denotes false negative. We calculated balanced accuracy, which is the mean of sensitivity and specificity. We generated ROC and precision-recall (PR) curves and calculated the ROC-AUC and PR-AUC, respectively.

### 2.4 Similarity-based screening

We developed GCNN models for transporters with sufficient data and implemented a similarity-based screening approach for the other transporters. The 2D chemical similarity approach uses atom connectivity/fingerprints and has been shown to perform well in retrieving related compounds (AbdulHameed et al., 2021). We evaluated the performance of the 2D similarity approach by examining whether the reference set of compounds for a particular target was able to identify chemicals already known to interact with the target when mixed with inactive compounds. We collected the reference set of compounds for 10 different targets from the DrugBank database and the corresponding external test dataset for each target from the ChEMBL database. We downloaded the largest available bioactivity data of the same type (IC_50_ or EC_50_ or K_i_) for each target. Most of the targets had IC_50_ values. We labelled compounds with activity values ≤1 μM as actives and ≥10 μM as inactives. Such binary thresholds are commonly used in cheminformatics studies (Chen et al., 2023). We pre-processed the data and removed duplicate compounds as well as overlapping compounds that were present in the reference set for that target. We performed screening for each target and calculated the performance using ROC-AUC values. Higher AUC values represented improved screening, whereas an AUC closer to 0.5 indicated that the approach was not able to separate active from inactive compounds.

In this work, we selected the known inhibitors or substrates of each transporter as the reference set of compounds for that transporter. Then, we calculated the similarity between each query compound and the reference set of compounds. Finally, we used the maximum similarity score (MAX) between them to represent the potential of the query compound to interact with the transporter.

### 2.5 Applicability domain

We previously developed an applicability domain method, the sum of distance-weighted contributions (SDC), that uses a distance-to-training set approach (Liu and Wallqvist, 2019). Basically, this class of approaches evaluates the chemical space for which the model will make reliable predictions. It uses the weighted distance between the query compound and all the molecules in the reference set to define the applicability domain (Liu and Wallqvist, 2019). SDC is defined as in Equation 5

$$\text{SDC}=\sum_{i=1}^{n}e^{-\frac{3{TD}_{i}}{1-{TD}_{i}}}$$ (5)

where TD_*i*_ represents the Tanimoto distance (TD) between a target molecule and the *i*th training molecule and *n* represents the total number of training molecules (Liu and Wallqvist, 2019). We calculated the TD between two molecules using the RDKit Morgan fingerprint with a radius of 2 (RDKit, 2024). The TD value between two compounds ranges from 0 to 1, and the higher the TD value, the lower the similarity between the compounds. We implemented the Python version of SDC and utilized it to define the applicability domain for all 24 transporter models developed so far. The script used to calculate SDC is provided on the GitHub page (https://github.com/bhsai/monstrous).

### 2.6 Web-tool development

The MONSTROUS web application runs on an Apache Tomcat server and utilizes a multi-tiered architecture consisting of a model inference engine, a front-end, a database, and a controller. At the core of the application is the model inference engine implemented as Python scripts that runs the GCNN models and the similarity-based screen. The user can submit up to 10,000 compounds at a time as SMILES through the front-end user interface (UI). The UI consists of forms for user inputs and pages to display model information and graphical outputs. The UI is built on the Flutter framework and is compatible with most modern web browsers and devices of varied screen sizes, including smartphones. The application back-end is a relational database hosted on PostgreSQL platform. The Java-based controller ties the front-end, the back-end, and the model inference engine together by providing pipes for efficient dataflow across the application. Additionally, the controller manages user authentication, session management, and job scheduling. Users can view the compounds they submitted and download the computed results for up to 2 weeks, after which users’ compounds and results are deleted from the database. None of user data, including compounds and results, is used to train new models or shared with anyone else at any point of time. The model inference engine along with the models developed in this work and the raw data used to train the models are available for download as a command-line tool at https://github.com/bhsai/monstrous. This command-line tool provides a batch screening option without any limit on the number of input query compounds. The MONSTROUS web application is publicly available at https://monstrous.bhsai.org/.

## 3 Results

The primary goal of this work was to develop a web-based tool to predict the potential of a chemical to interact with transporters that are recommended by regulatory agencies for screening during the drug development process. To achieve this, first, we surveyed and collected the inhibitor and substrate data for these transporters.

### 3.1 Transporter bioactivity data collection

**FIGURE 2:**
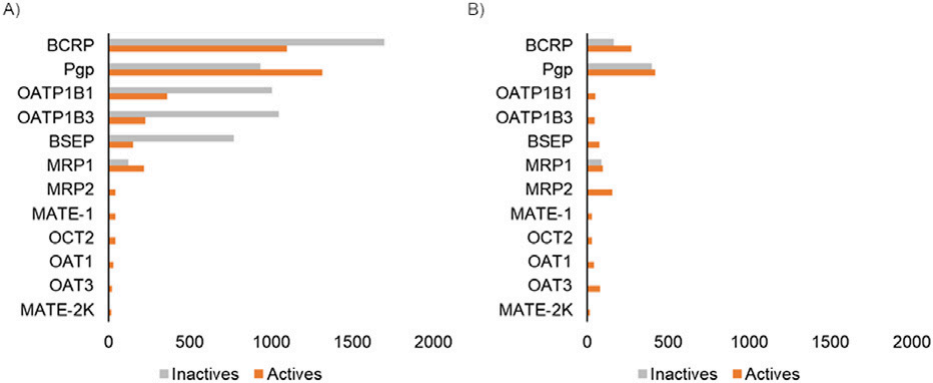
Count of bioactivity data associated with the 12 transporters used in this study. **(A)** Data associated with transporter inhibitors. **(B)** Data associated with transporter substrates. Each bar represents the number of compounds with experimentally measured activity for the corresponding transporter.

We collected publicly available datasets associated with 12 transporters recommended for screening by regulatory agencies, including five transporters from the ABC family and seven transporters from the SLC family (Table 1). Overall, BCRP and Pgp, which are known to be associated with cancer-induced multi-drug resistance, had the greatest data availability, with more than 2,000 compounds with associated transporter inhibition data (Figure 2). The amount of available data associated with transporter substrates was significantly lower than that for inhibitors (Figure 2). BSEP is a well-known transporter associated with bile transport, and its inhibition leads to cholestasis. While we were able to collect some known substrates for this transporter, we were not able to obtain compounds that are non-substrates. BCRP and Pgp had the largest number of compounds with substrate activity data. Overall, the sparse publicly available bioactivity data for transporters highlights the challenge in creating a computational tool for rapid transporter liability screening. In this work, to address this bottleneck, we developed machine learning models for those transporters with sufficient bioactivity data and implemented a 2D similarity-based screening approach for the others. Table 1 lists the 12 transporters used in this work along with information on whether the web tool uses machine learning models or the similarity-based approach to make predictions on new query compounds. Our final set includes 24 transporter models, nine of which are GCNN models and 15 of which utilize the similarity-based screening approach.

**TABLE 1:** List of transporters included in the MONSTROUS web tool along with their transporter family, commonly used name, gene symbol, and type of screening approach implemented.

No	Class		Transporters	Gene	Screening approach	
					Inhibitors	Substrates
1	ABC		Pgp	ABCB1	GCNN	GCNN
2			BCRP	ABCG2	GCNN	GCNN
3			MRP1	ABCC1	GCNN	GCNN
4			BSEP	ABCB11	GCNN	Similarity
5			MRP2	ABCC2	Similarity	Similarity
6	SLC	SLCO	OATP1B1	SLCO1B1	GCNN	Similarity
7			OATP1B3	SLCO1B3	GCNN	Similarity
8		SLC22	OAT1	SLC22A6	Similarity	Similarity
9			OAT3	SLC22A8	Similarity	Similarity
10			OCT2	SLC22A2	Similarity	Similarity
11		SLC47	MATE-1	SLC47A1	Similarity	Similarity
12			MATE-2K	SLC47A2	Similarity	Similarity

### 3.2 GCNN models

**FIGURE 3:**
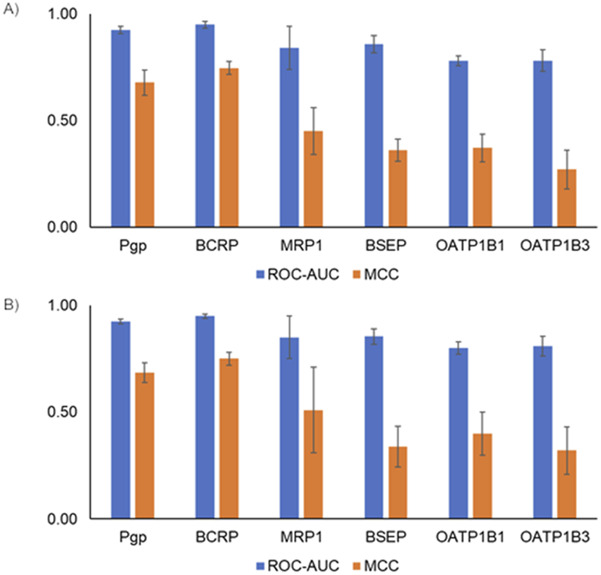
Performance metrics from cross-validation analyses for the transporter inhibitor models. **(A)** Five-fold cross-validation analysis. **(B)** 10-fold cross-validation analysis. ROC-AUC, receiver operating characteristic area under the curve; MCC, Matthews correlation coefficient.

**FIGURE 4:**
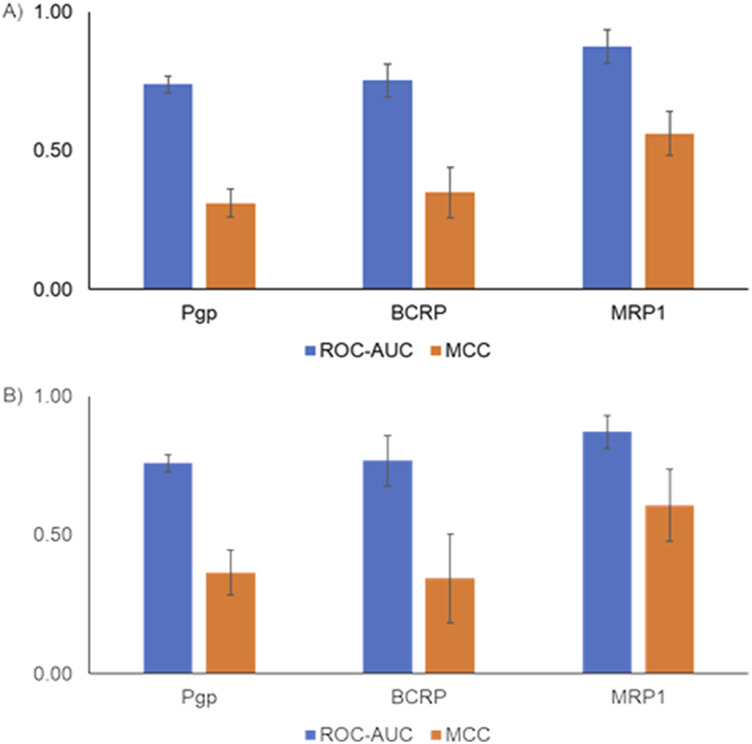
Performance metrics from cross-validation analyses for the transporter substrate models. **(A)** Five-fold cross-validation analysis. **(B)** 10-fold cross-validation analysis. ROC-AUC, receiver operating characteristic area under the curve; MCC, Matthews correlation coefficient.

Machine learning models are widely used for predicting various absorption, distribution, metabolism, excretion, and toxicity endpoints (Wang et al., 2021). Typically, to develop such models, the structure of the compounds is represented using fingerprints or molecular properties. Fingerprints capture the presence of predefined sets of chemical substructures/functional groups to represent the chemical structure. More recently, GCNN-based approaches provide an alternative approach as they allow us to learn the representation of chemical structures in an automated manner (Chuang et al., 2020). Others have performed a detailed analysis of benchmark datasets and have shown that GCNNs perform better than other machine learning approaches for predicting the bioactivity of compounds (Yang et al., 2019). Although the use of GCNNs has been reported to be a powerful approach, so far they have not been widely used in predicting transporter interactions. In this work, we developed six transporter inhibitor models and three transporter substrate models using the GCNN approach. Our inhibitor GCNN models include four ABC transporters (Pgp, BCRP, MRP1, BSEP) and two SLC transporters (OATP1B1, OATP1B3). We developed three substrate GCNN models for Pgp, BCRP, and MRP1. We used five-fold and 10-fold cross-validation analyses and obtained performance evaluation metrics for these models. Figure 3 provides the five-fold and 10-fold cross-validation performance metrics for the inhibitor models. All six transporter inhibitor models had ROC-AUC >0.7 and MCC >0.2 in both cross-validations. The ROC-AUC values in the 10-fold cross-validation analysis are on par with the performance reported for the Vienna LiverTox web tool. As mentioned in the Introduction, Vienna LiverTox is a web-based tool for screening transporter interactions related to liver injury. Figure 4 provides the five-fold and 10-fold cross-validation performance metrics for the substrate models. Similar to the inhibitor models, all three substrate models had ROC-AUC >0.7 and MCC >0.2 in both cross-validations.

**FIGURE 5:**
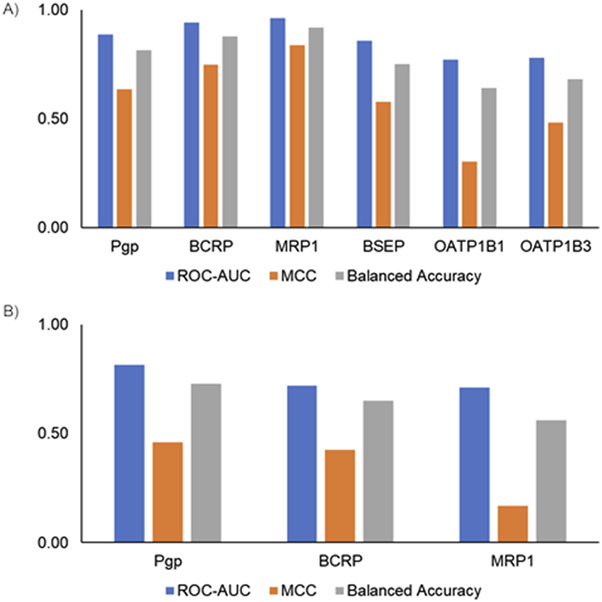
Performance metrics using a single scaffold-split test set for **(A)** inhibitor models and **(B)** substrate models. ROC-AUC, receiver operating characteristic area under the curve; MCC, Matthews correlation coefficient.

Finally, we evaluated model performance by creating an external test set using the scaffold-split approach. Scaffold split-based analysis provides an alternate way to evaluate model performance. Figure 5 shows the scaffold split-based evaluation results for the inhibitor and substrate models. All the inhibitor and substrate models demonstrated reasonable performance, with average ROC-AUC >0.7 and balanced accuracy >0.5. Among the inhibitor models, the MRP1 and BCRP models exhibited the highest balanced accuracy, whereas the OATP1B1 and OATP1B3 models had the lowest, with values closer to 65%. Similarly, for the substrate models, the Pgp model achieved the highest balanced accuracy, while the MRP1 model showed the lowest. The inhibitor models overall performed slightly better than the substrate models, likely due to the availability of more training data for inhibitors. The final models were built using all available data and integrated into the MONSTROUS web tool to facilitate easy access. Additionally, we implemented the SDC approach to define the applicability domain and provide clear guidance on where predictions are most reliable.

Overall, we utilized GCNN, a well-established approach for property prediction, to develop models for transporters recommended by regulatory agencies and integrated them into an easy-to-use web interface. As a limitation, we acknowledge that this study primarily focused on tool development and has exclusively used the GCNN approach. Future work will explore comparisons with other available methods to further enhance the tool’s predictive performance.

### 3.3 Similarity-based screening approach

**FIGURE 6:**
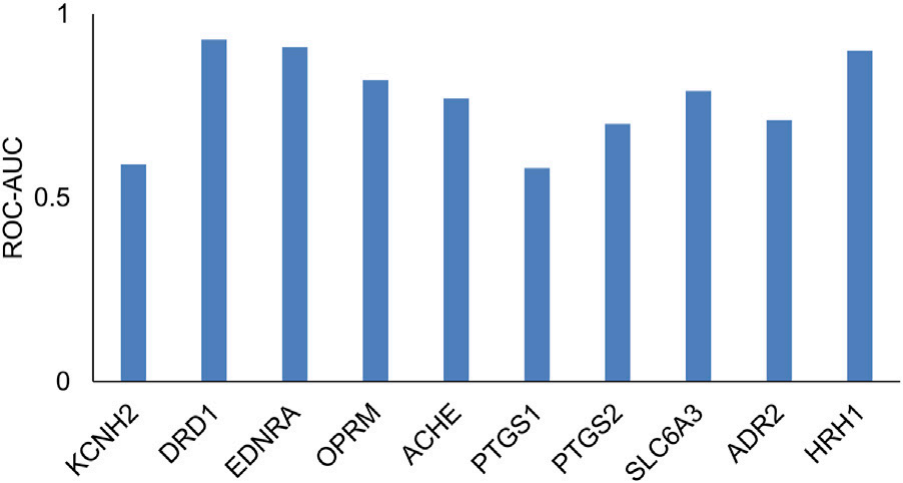
Receiver operating characteristic area under the curve (ROC-AUC) values, which evaluated the performance of the 2D similarity approach (via MAX Tanimoto scores) to retrieve hits in an external test set across the 10 targets.

Many transporters do not have sufficient data, particularly with regard to compounds that are inactive as inhibitors or substrates. In such cases, it is not possible to develop machine learning models. In order to provide a comprehensive tool that covers all regulatory-relevant transporters, we implemented a 2D similarity-based screening tool. First, we tested whether such an approach is reasonable using a test evaluation of 10 targets with sufficient data. Figure 6 and Supplementary Table S3 provide a list of the 10 targets evaluated. We evaluated the ability of a small set of known reference compounds (active compounds) for these 10 targets to screen an external set of active and inactive compounds from ChEMBL using the 2D similarity approach. We calculated the MAX Tanimoto scores between the reference and query compounds. Figure 6 shows the ROC-AUC values for these targets. We found that eight of the 10 targets had ROC-AUC >0.6, indicating that the 2D similarity approach was able to retrieve active compounds from inactive compounds and can serve as a screening tool for transporters with low publicly available data. As highlighted in Table 1, we implemented this 2D similarity approach in inhibitor mode for six transporters (MRP2, OAT1, OAT3, OCT2, MATE-1, MATE-2K) and in substrate mode for nine transporters (MRP2, BSEP, OATP1B1, OATP1B3, OAT1, OAT3, OCT2, MATE-1, MATE-2K). Similarity-based models rely on the presence of structurally similar molecules in the reference set, which may lead to less reliable predictions for novel or unique chemical scaffolds not represented in the reference set. However, the hybrid approach of combining GCNN and similarity-based models is practically useful, as it enables users to screen against all 12 transporters within a single tool. As more data become available, we plan to upgrade the models in future versions of the tool to enhance prediction accuracy.

### 3.4 MONSTROUS web interface

One of the main goals of this work was to create an easy-to-use, publicly available, web-based computational tool for rapid screening and prediction of the potential of chemicals to interact with transporters. Here, we created one such web interface that allows easy access to the deep learning models and similarity-based screening tools as a means to rapidly identify chemicals with potential to interact with transporters either as an inhibitor or a substrate.

**FIGURE 7:**
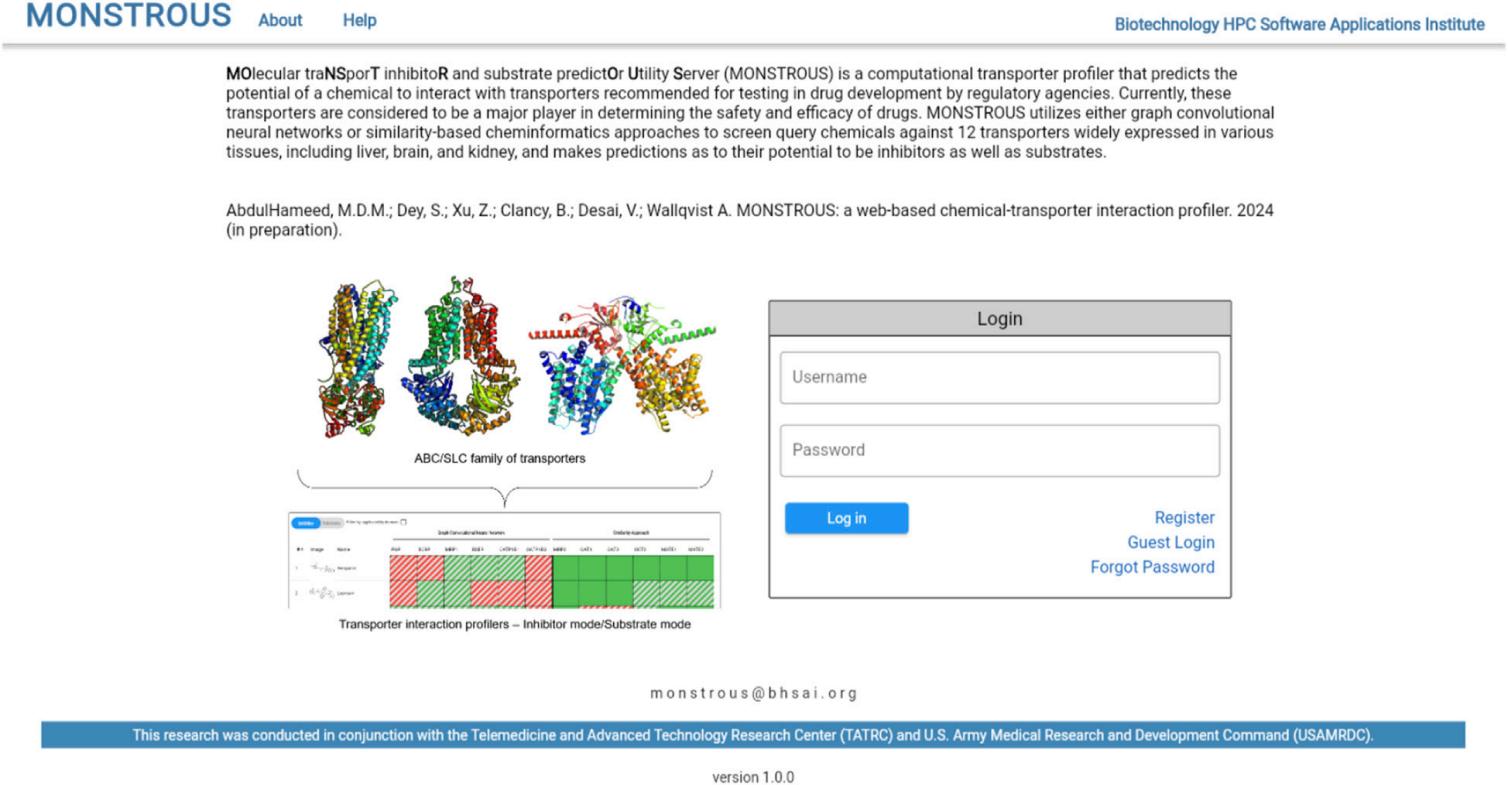
Login page for the web-based user interface (https://monstrous.bhsai.org/). The system supports registration of users as well as limited guest accounts to explore the system.

**FIGURE 8:**
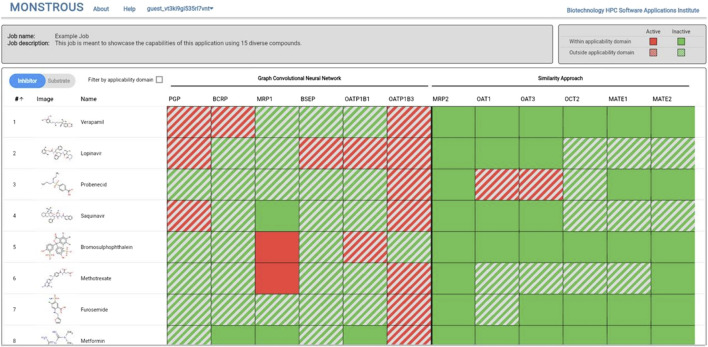
MONSTROUS overall results page for a series of compounds displaying input structures and names and color-coded assessments of the likelihood of each chemical to interact with particular transporters in either the inhibitor mode or substrate mode. Predictions that are outside of the applicability domain are shown by striped colors. The results can be downloaded and stored by the user.

The MONSTROUS website is publicly available online at https://monstrous.bhsai.org/. The login page (Figure 7) allows users to create a new account or log in to an existing or guest account. After login, users can submit query compounds in SMILES format by typing directly, uploading a CSV file, or using the Marvin chemical drawing tool (Chemaxon, Boston, MA). Job status can be monitored on the homepage. Figure 8 shows a snapshot of the output, which provides separate views for transporter inhibition and substrate predictions as well as the ability to toggle between them. Users also have the option to view the results with the applicability domain. The output is color-coded for easy interpretation: red indicates active (inhibitor or substrate) and green indicates inactive. The predictions outside the applicability domain are shown in striped red and green cells. Selecting the applicability domain option will show these output cells in gray. Users have the option to download the results as an Excel heatmap or as raw data. Both options provide a table with separate sheets for inhibitor and substrate predictions, with and without the applicability domain. The heatmap is color-coded to represent the potential of query chemicals to interact with the transporter (red) or to have no interaction (green).

In addition to the web application, users with computational expertise can utilize the command-line version of the tool at https://github.com/bhsai/monstrous. Users can download, install, and locally run this command-line tool on their infrastructure. The web application allows batch screening of up to 10,000 compounds at a time, while the command-line version provides batch screening with no limit on the number of input query compounds.

### 3.5 Example analysis

**FIGURE 9:**
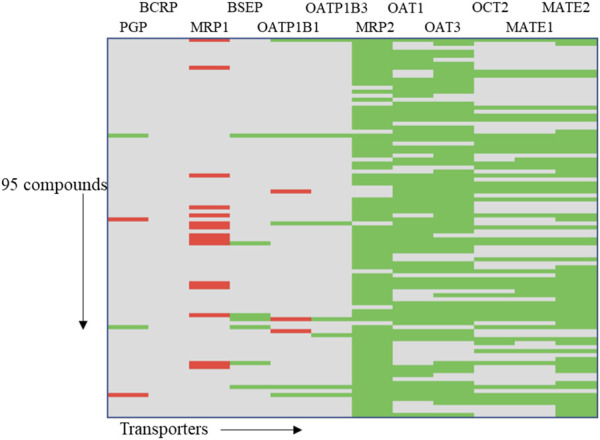
Results of an example analysis of 95 kinase inhibitors for transporter inhibition using MONSTROUS. We generated a chemical-transporter interaction profile for 95 query chemicals across 12 transporters. Red represents predicted interactions, green represents lack of interaction, and gray represents that the prediction is outside of the applicability domain of the model. Our results show that these well-characterized kinase inhibitors have the potential for interaction with transporters such as MRP1, OATP1B1, and PGP.

In order to demonstrate the utility of MONSTROUS, we conducted an example analysis using a dataset of 95 well-characterized kinase inhibitors (Supplementary Table S2). These compounds were part of the IDG-DREAM Drug-Kinase Binding Prediction Challenge, where their interactions with 295 kinases were experimentally profiled and made publicly available (Cichońska et al., 2021). FDA-approved kinase inhibitors, such as imatinib and gefitinib, are known to interact with ABC transporters, highlighting the importance of evaluating transporter liability. While the 95 compounds from the IDG-DREAM challenge have been extensively studied for off-target kinase interactions, their transporter liability remains unknown. We anticipate that *in silico* tools like MONSTROUS will help bridge this gap. Figure 9 shows the prediction results for these 95 compounds using the MONSTROUS tool. Our predictions indicate that of the 95 compounds, 13 are potential inhibitors of MRP1, three inhibit OATP1B1, and 11 are substrates for BCRP. This analysis highlights how MONSTROUS can provide insights on potential transporter liability for query compounds of interest.

## 4 Discussion

Chemical transporter interaction has varied effects, from adverse health effects to altered pharmacokinetic profiles to unwanted drug-drug interactions. Regulatory agencies now recommend screening of new molecular entities against transporters during drug development. Computational tools allow rapid screening and earlier identification of such chemical transporter interactions. In this work, we created one such publicly available and easy-to-use web-based tool: MONSTROUS. This tool allows rapid screening and prediction of the potential of chemicals to interact with transporters recommended by regulatory agencies.

Chemical transporter interactions have two facets: 1) the chemical can act as a substrate and the transporter interaction facilitates its movement into or out of the cell or 2) the chemical can act as an inhibitor and prevent the function of the transporter. The substrate and inhibitor potentials are studied using different types of assays, and most of the publicly available data are associated with studying transporter inhibition since this assay is more amenable to the high-throughput screening format. This scenario is reflected in the publicly available data for transporters (Figure 2). For example, the transporter with the most available bioactivity data, i.e., Pgp, has 2,000 chemicals with inhibition data but only 900 with substrate data. Also, even for well-known transporters such as BSEP, we could not find any publicly available non-substrate data, which limits our ability to develop machine learning models. We reasoned that even having a tool that allows for the capture of existing knowledge about these transporters will be practically useful for researchers to understand the transporter liability of their compounds of interest. Hence, we developed the MONSTROUS tool with both deep learning models as well as using the 2D similarity-based screening approach. This allowed us to develop a tool that makes predictions for all the transporters recommended by regulatory agencies for both inhibition and substrate potential.

One of the key Organisation for Economic Co-operation and Development (OECD) principles for machine learning models is that they should have a defined applicability domain, which indicates whether the model can reliably predict the activity of a new compound. We previously developed the SDC approach, which considers the contributions of all training molecules in gauging the reliability of a prediction. In our previously reported evaluation, SDC outperformed other commonly used applicability domain methods. Hence, we implemented an SDC-based applicability domain for all the models.

Our performance evaluation of the developed models shows that they perform on par with previously reported machine learning models for transporters. We also showed that the MAX Tanimoto similarity approach performs well for retrieving the actives from an external test set for 10 tested targets. We implemented all these approaches and used them to develop a comprehensive platform that makes use of publicly available data for transporters recommended by regulators and enables rapid screening for predicting potential transporter interactions for query chemicals. We made this resource publicly available via a web-user interface. Overall, our MONSTROUS tool will be useful in prioritizing hits and understanding transporter liability in early drug discovery projects.

## Data Availability

The original contributions presented in the study are included in the article/Supplementary Material, further inquiries can be directed to the corresponding authors.

## References

[B1] AbdulHameed M. D. Ippolito D. L. Wallqvist A. (2016). Predicting rat and human pregnane X receptor activators using bayesian classification models. Chem. Res. Toxicol. 29 (10), 1729–1740. 10.1021/acs.chemrestox.6b00227 27603675

[B2] AbdulHameed M. D. M. Liu R. Schyman P. Sachs D. Xu Z. Desai V. (2021). ToxProfiler: toxicity-target profiler based on chemical similarity. Comput. Toxicol. 18, 100162. 10.1016/j.comtox.2021.100162

[B3] AbdulHameed M. D. M. Liu R. Wallqvist A. (2023). Using a graph convolutional neural network model to identify bile salt export pump inhibitors. ACS Omega 8 (24), 21853–21861. 10.1021/acsomega.3c01583 37360478 PMC10286257

[B4] Aniceto N. Freitas A. A. Bender A. Ghafourian T. (2016). Simultaneous prediction of four ATP-binding cassette transporters' substrates using multi-label QSAR. Mol. Inf. 35 (10), 514–528. 10.1002/minf.201600036 27582431

[B5] Bellettato C. M. Scarpa M. (2018). Possible strategies to cross the blood-brain barrier. Ital. J. Pediatr. 44 (Suppl. 2), 131. 10.1186/s13052-018-0563-0 30442184 PMC6238258

[B6] Bento A. P. Hersey A. Felix E. Landrum G. Gaulton A. Atkinson F. (2020). An open source chemical structure curation pipeline using RDKit. J. Cheminform 12 (1), 51. 10.1186/s13321-020-00456-1 33431044 PMC7458899

[B7] Chen Y. Yu X. Li W. Tang Y. Liu G. (2023). *In silico* prediction of hERG blockers using machine learning and deep learning approaches. J. Appl. Toxicol. 43 (10), 1462–1475. 10.1002/jat.4477 37093028

[B8] Chuang K. V. Gunsalus L. M. Keiser M. J. (2020). Learning molecular representations for medicinal chemistry. J. Med. Chem. 63 (16), 8705–8722. 10.1021/acs.jmedchem.0c00385 32366098

[B59] Cichońska A. Ravikumar B. Allaway R. J. Wan F. Park S. Isayev O. (2021). Crowdsourced mapping of unexplored target space of kinase inhibitors. Nat. Commun. 12 (1), 3307. 10.1038/s41467-021-23165-1 34083538 PMC8175708

[B9] Ciuta A. D. Nosol K. Kowal J. Mukherjee S. Ramirez A. S. Stieger B. (2023). Structure of human drug transporters OATP1B1 and OATP1B3. Nat. Commun. 14 (1), 5774. 10.1038/s41467-023-41552-8 37723174 PMC10507018

[B10] Ekins S. Kim R. B. Leake B. F. Dantzig A. H. Schuetz E. G. Lan L. B. (2002). Application of three-dimensional quantitative structure-activity relationships of P-glycoprotein inhibitors and substrates. Mol. Pharmacol. 61 (5), 974–981. 10.1124/mol.61.5.974 11961114

[B11] Galetin A. Brouwer K. L. R. Tweedie D. Yoshida K. Sjostedt N. Aleksunes L. (2024). Membrane transporters in drug development and as determinants of precision medicine. Nat. Rev. Drug Discov. 23 (4), 255–280. 10.1038/s41573-023-00877-1 38267543 PMC11464068

[B12] Giacomini K. M. Yee S. W. Koleske M. L. Zou L. Matsson P. Chen E. C. (2022). New and emerging research on solute carrier and ATP binding cassette transporters in drug discovery and development: outlook from the International Transporter Consortium. Clin. Pharmacol. Ther. 112 (3), 540–561. 10.1002/cpt.2627 35488474 PMC9398938

[B13] Gilson M. K. Liu T. Baitaluk M. Nicola G. Hwang L. Chong J. (2016). BindingDB in 2015: a public database for medicinal chemistry, computational chemistry and systems pharmacology. Nucleic Acids Res. 44 (D1), D1045–D1053. 10.1093/nar/gkv1072 26481362 PMC4702793

[B14] Gordon L. A. Kumar P. Brooks K. M. Kellogg A. McManus M. Alfaro R. M. (2016). Antiretroviral boosting agent cobicistat increases the pharmacokinetic exposure and anticoagulant effect of dabigatran in HIV-negative healthy volunteers. Circulation 134 (23), 1909–1911. 10.1161/CIRCULATIONAHA.116.025257 27920076 PMC5145004

[B15] Heid E. Green W. H. (2022). Machine learning of reaction properties via learned representations of the condensed graph of reaction. J. Chem. Inf. Model. 62 (9), 2101–2110. 10.1021/acs.jcim.1c00975 34734699 PMC9092344

[B16] Heid E. Greenman K. P. Chung Y. Li S. C. Graff D. E. Vermeire F. H. (2024). Chemprop: a machine learning package for chemical property prediction. J. Chem. Inf. Model. 64 (1), 9–17. 10.1021/acs.jcim.3c01250 38147829 PMC10777403

[B17] International Transporter Consortium, Giacomini K. M. Huang S. M. Tweedie D. J. Benet L. Z. Brouwer K. L. (2010). Membrane transporters in drug development. Nat. Rev. Drug Discov. 9 (3), 215–236. 10.1038/nrd3028 20190787 PMC3326076

[B18] Iorio A. L. Ros M. Fantappie O. Lucchesi M. Facchini L. Stival A. (2016). Blood-brain barrier and breast cancer resistance protein: a limit to the therapy of CNS tumors and neurodegenerative diseases. Anticancer Agents Med. Chem. 16 (7), 810–815. 10.2174/1871520616666151120121928 26584727 PMC4997940

[B19] Jiang D. Lei T. Wang Z. Shen C. Cao D. Hou T. (2020). ADMET evaluation in drug discovery. 20. Prediction of breast cancer resistance protein inhibition through machine learning. J. Cheminform 12 (1), 16. 10.1186/s13321-020-00421-y 33430990 PMC7059329

[B20] Kohler S. C. Wiese M. (2015). HM30181 derivatives as novel potent and selective inhibitors of the breast cancer resistance protein (BCRP/ABCG2). J. Med. Chem. 58 (9), 3910–3921. 10.1021/acs.jmedchem.5b00188 25855895

[B21] Kong X. Lin K. Wu G. Tao X. Zhai X. Lv L. (2023). Machine learning techniques applied to the study of drug transporters. Molecules 28 (16), 5936. 10.3390/molecules28165936 37630188 PMC10459831

[B22] Kotsampasakou E. Brenner S. Jager W. Ecker G. F. (2015). Identification of novel inhibitors of organic anion transporting polypeptides 1B1 and 1B3 (OATP1B1 and OATP1B3) using a consensus vote of six classification models. Mol. Pharm. 12 (12), 4395–4404. 10.1021/acs.molpharmaceut.5b00583 26469880 PMC4674819

[B23] Kotsampasakou E. Ecker G. F. (2017). Predicting drug-induced cholestasis with the help of hepatic transporters-an *in silico* modeling approach. J. Chem. Inf. Model. 57 (3), 608–615. 10.1021/acs.jcim.6b00518 28166633 PMC5411109

[B24] Kwon Y. Park S. Lee J. Kang J. Lee H. J. Kim W. (2023). BEAR: a novel virtual screening method based on large-scale bioactivity data. J. Chem. Inf. Model. 63 (5), 1429–1437. 10.1021/acs.jcim.2c01300 36821004

[B25] Lane T. R. Urbina F. Zhang X. Fye M. Gerlach J. Wright S. H. (2022). Machine learning models identify new inhibitors for human OATP1B1. Mol. Pharm. 19 (11), 4320–4332. 10.1021/acs.molpharmaceut.2c00662 36269563 PMC9873312

[B26] Li H. Zhang S. L. Jia Y. H. Li Q. Feng Z. W. Zhang S. D. (2023). Imidazo[1,2-a]Pyridine derivatives as novel dual-target inhibitors of ABCB1 and ABCG2 for reversing multidrug resistance. J. Med. Chem. 66 (4), 2804–2831. 10.1021/acs.jmedchem.2c01862 36780419

[B27] Liu H. Sahi J. (2016). Role of hepatic drug transporters in drug development. J. Clin. Pharmacol. 56 (Suppl. 7), S11–S22. 10.1002/jcph.703 27385168

[B28] Liu K. Sun X. Jia L. Ma J. Xing H. Wu J. (2019). Chemi-net: a molecular graph convolutional network for accurate drug property prediction. Int. J. Mol. Sci. 20 (14), 3389. 10.3390/ijms20143389 31295892 PMC6678642

[B29] Liu R. AbdulHameed M. D. M. Kumar K. Yu X. Wallqvist A. Reifman J. (2017). Data-driven prediction of adverse drug reactions induced by drug-drug interactions. BMC Pharmacol. Toxicol. 18 (1), 44. 10.1186/s40360-017-0153-6 28595649 PMC5465578

[B30] Liu R. Wallqvist A. (2019). Molecular similarity-based domain applicability metric efficiently identifies out-of-domain compounds. J. Chem. Inf. Model. 59 (1), 181–189. 10.1021/acs.jcim.8b00597 30404432

[B31] Luechtefeld T. Rowlands C. Hartung T. (2018). Big-data and machine learning to revamp computational toxicology and its use in risk assessment. Toxicol. Res. (Camb) 7 (5), 732–744. 10.1039/c8tx00051d 30310652 PMC6116175

[B32] Mak L. Marcus D. Howlett A. Yarova G. Duchateau G. Klaffke W. (2015). Metrabase: a cheminformatics and bioinformatics database for small molecule transporter data analysis and (Q)SAR modeling. J. Cheminform 7, 31. 10.1186/s13321-015-0083-5 26106450 PMC4477067

[B33] McLoughlin K. S. Jeong C. G. Sweitzer T. D. Minnich A. J. Tse M. J. Bennion B. J. (2021). Machine learning models to predict inhibition of the bile salt export pump. J. Chem. Inf. Model. 61 (2), 587–602. 10.1021/acs.jcim.0c00950 33502191

[B34] Montanari F. Ecker G. F. (2015). Prediction of drug-ABC-transporter interaction--Recent advances and future challenges. Adv. Drug Deliv. Rev. 86, 17–26. 10.1016/j.addr.2015.03.001 25769815 PMC6422311

[B35] Montanari F. Knasmuller B. Kohlbacher S. Hillisch C. Baierova C. Grandits M. (2019). Vienna LiverTox workspace-A set of machine learning models for prediction of interactions profiles of small molecules with transporters relevant for regulatory agencies. Front. Chem. 7, 899. 10.3389/fchem.2019.00899 31998690 PMC6966498

[B36] Montanari F. Zdrazil B. (2017). How open data shapes *in silico* transporter modeling. Molecules 22 (3), 422. 10.3390/molecules22030422 28272367 PMC5553104

[B37] Munagala S. Sirasani G. Kokkonda P. Phadke M. Krynetskaia N. Lu P. (2014). Synthesis and evaluation of Strychnos alkaloids as MDR reversal agents for cancer cell eradication. Bioorg Med. Chem. 22 (3), 1148–1155. 10.1016/j.bmc.2013.12.022 24405813

[B38] Namasivayam V. Silbermann K. Wiese M. Pahnke J. Stefan S. M. (2021). C@PA: computer-aided pattern analysis to predict multitarget ABC transporter inhibitors. J. Med. Chem. 64 (6), 3350–3366. 10.1021/acs.jmedchem.0c02199 33724808 PMC8041314

[B39] Nigam A. K. Momper J. D. Ojha A. A. Nigam S. K. (2024). Distinguishing molecular properties of OAT, OATP, and MRP drug substrates by machine learning. Pharmaceutics 16 (5), 592. 10.3390/pharmaceutics16050592 38794254 PMC11125978

[B40] Nigam S. K. (2015). What do drug transporters really do? Nat. Rev. Drug Discov. 14 (1), 29–44. 10.1038/nrd4461 25475361 PMC4750486

[B41] Pan Y. Chothe P. P. Swaan P. W. (2013). Identification of novel breast cancer resistance protein (BCRP) inhibitors by virtual screening. Mol. Pharm. 10 (4), 1236–1248. 10.1021/mp300547h 23418667

[B42] Pena-Solorzano D. Stark S. A. Konig B. Sierra C. A. Ochoa-Puentes C. (2017). ABCG2/BCRP: specific and nonspecific modulators. Med. Res. Rev. 37 (5), 987–1050. 10.1002/med.21428 28005280

[B43] Pinto M. Trauner M. Ecker G. F. (2012). An *in silico* classification model for putative ABCC2 substrates. Mol. Inf. 31 (8), 547–553. 10.1002/minf.201200049 PMC3505902 23198001

[B44] Poongavanam V. Haider N. Ecker G. F. (2012). Fingerprint-based *in silico* models for the prediction of P-glycoprotein substrates and inhibitors. Bioorg Med. Chem. 20 (18), 5388–5395. 10.1016/j.bmc.2012.03.045 22595422 PMC3445814

[B45] Rankovic Z. (2015). CNS drug design: balancing physicochemical properties for optimal brain exposure. J. Med. Chem. 58 (6), 2584–2608. 10.1021/jm501535r 25494650

[B46] RDKit (2024). Open-source cheminformatics. Available at: https://www.rdkit.org.

[B47] Sajid A. Rahman H. Ambudkar S. V. (2023). Advances in the structure, mechanism and targeting of chemoresistance-linked ABC transporters. Nat. Rev. Cancer 23 (11), 762–779. 10.1038/s41568-023-00612-3 37714963

[B48] Schlessinger A. Welch M. A. van Vlijmen H. Korzekwa K. Swaan P. W. Matsson P. (2018). Molecular modeling of drug-transporter interactions-an International Transporter Consortium perspective. Clin. Pharmacol. Ther. 104 (5), 818–835. 10.1002/cpt.1174 29981151 PMC6197929

[B49] Schyman P. Liu R. Wallqvist A. (2016). Using the variable-nearest neighbor method to identify P-glycoprotein substrates and inhibitors. ACS Omega 1 (5), 923–929. 10.1021/acsomega.6b00247 30023496 PMC6044698

[B50] Sedykh A. Fourches D. Duan J. Hucke O. Garneau M. Zhu H. (2013). Human intestinal transporter database: QSAR modeling and virtual profiling of drug uptake, efflux and interactions. Pharm. Res. 30 (4), 996–1007. 10.1007/s11095-012-0935-x 23269503 PMC3596480

[B51] Shaikh N. Sharma M. Garg P. (2017). Selective fusion of heterogeneous classifiers for predicting substrates of membrane transporters. J. Chem. Inf. Model. 57 (3), 594–607. 10.1021/acs.jcim.6b00508 28228010

[B52] Silbermann K. Li J. Namasivayam V. Baltes F. Bendas G. Stefan S. M. (2020). Superior pyrimidine derivatives as selective ABCG2 inhibitors and broad-spectrum ABCB1, ABCC1, and ABCG2 antagonists. J. Med. Chem. 63 (18), 10412–10432. 10.1021/acs.jmedchem.0c00961 32787102

[B53] Turkova A. Jain S. Zdrazil B. (2019). Integrative data mining, scaffold analysis, and sequential binary classification models for exploring ligand profiles of hepatic organic anion transporting polypeptides. J. Chem. Inf. Model. 59 (5), 1811–1825. 10.1021/acs.jcim.8b00466 30372058 PMC6541895

[B54] Turkova A. Zdrazil B. (2019). Current advances in studying clinically relevant transporters of the solute carrier (SLC) family by connecting computational modeling and data science. Comput. Struct. Biotechnol. J. 17, 390–405. 10.1016/j.csbj.2019.03.002 30976382 PMC6438991

[B55] Vinken M. Landesmann B. Goumenou M. Vinken S. Shah I. Jaeschke H. (2013). Development of an adverse outcome pathway from drug-mediated bile salt export pump inhibition to cholestatic liver injury. Toxicol. Sci. 136 (1), 97–106. 10.1093/toxsci/kft177 23945500

[B56] Wang M. W. H. Goodman J. M. Allen T. E. H. (2021). Machine learning in predictive toxicology: recent applications and future directions for classification models. Chem. Res. Toxicol. 34 (2), 217–239. 10.1021/acs.chemrestox.0c00316 33356168

[B57] Yang K. Swanson K. Jin W. Coley C. Eiden P. Gao H. (2019). Analyzing learned molecular representations for property prediction. J. Chem. Inf. Model. 59 (8), 3370–3388. 10.1021/acs.jcim.9b00237 31361484 PMC6727618

[B58] Zdrazil B. Felix E. Hunter F. Manners E. J. Blackshaw J. Corbett S. (2024). The ChEMBL Database in 2023: a drug discovery platform spanning multiple bioactivity data types and time periods. Nucleic Acids Res. 52 (D1), D1180–D1192. 10.1093/nar/gkad1004 37933841 PMC10767899

